# Candidate gene association studies: a comprehensive guide to useful *in silico* tools

**DOI:** 10.1186/1471-2156-14-39

**Published:** 2013-05-09

**Authors:** Radhika Patnala, Judith Clements, Jyotsna Batra

**Affiliations:** 1 Australian Prostate Cancer Research Centre – Queensland, Institute of Health and Biomedical Innovation, Queensland University of Technology, Brisbane, QLD 4059, Australia

**Keywords:** Candidate gene, SNP, LD, *In-silico*, Association studies, Cancer

## Abstract

The candidate gene approach has been a pioneer in the field of genetic epidemiology, identifying risk alleles and their association with clinical traits. With the advent of rapidly changing technology, there has been an explosion of *in silico* tools available to researchers, giving them fast, efficient resources and reliable strategies important to find casual gene variants for candidate or genome wide association studies (GWAS). In this review, following a description of candidate gene prioritisation, we summarise the approaches to single nucleotide polymorphism (SNP) prioritisation and discuss the tools available to assess functional relevance of the risk variant with consideration to its genomic location. The strategy and the tools discussed are applicable to any study investigating genetic risk factors associated with a particular disease. Some of the tools are also applicable for the functional validation of variants relevant to the era of GWAS and next generation sequencing (NGS).

## Review

### Introduction

Candidate gene studies have been at the forefront of genetic association studies i.e. identifying risk variants associated with a particular disease. Candidate gene studies are relatively cheap and quick to perform, and are focused on the selection of genes that have been in some way related to the disease previously and thus come with prior knowledge about gene function. The candidate gene approach begins with selection of a putative candidate gene based on its relevance in the mechanism of the disease (trait) being investigated [1]. This is followed by assessing and selecting polymorphisms, usually the tag Single NucleotidePolymorphim (SNPs) (described later in this review) and/or having a functional consequence, either by affecting gene regulation or its protein product [1,2]. Finally, the gene variant is verified for disease (trait) association by observing its occurrence in random test subjects (cases) having the disease and the selected control subjects which do not; and is then evaluated for its association with disease prognosis and diagnosis and its future potential as a biomarker. This makes the knowledge derived from candidate gene studies valuable and clinically relevant as a potential disease diagnostic tool and for personalised medicine initiatives in future treatments of genetic disorders [3].

Candidate gene association studies have been criticised on some aspects, but these can be duly overcome by the range of new tools and resources developed to this end. One such aspect is non-replication of results. One of the major issues for non-replication of the results involves population stratification, which can easily be circumvented by considering a replication study using an independent and random cohort of test and control populations, which reduces the chance of occurrence of a similar admixture showing similar patterns of variations [4]. The many collaborative projects taking place in recent years, such as the international HapMap project (http://hapmap.ncbi.nlm.nih.gov/) [5] and 1000 genomes project (http://www.1000genomes.org/) [6], provide researchers with allele frequencies of SNPs and their correlation pattern (haplotypes) to analyse population stratification before pooling data from different populations. Another aspect is the mild uncertainty about if the results portray disease susceptibility of a common variant, or do they just represent certain ancestral differences existing by chance between the mixes of test or control populations. Additionally, the multiple comparisons issue due to accounting for the same SNP in various tests can lead to false discovery rates. This can be addressed in two ways, first by computing Bonferroni adjustments of the significance criterion (alpha) according to the number of genes/SNPs/haplotypes (described later in this review) examined and second by performing permutation analysis of the association with allelic variation in the associating haplotype block. Although some argue that candidate gene studies must still meet statistical criteria for genome-wide significance, such a conservative threshold seems overly stringent, particularly in the context of a disorder with no (known) major gene effects. One of the other reasons for identifying a number of false positive findings could involve systemic genotyping errors, lack of statistical power due to smaller samples. In other instances, false negative findings (type II error) could be the reason for non-replication [7,8]. False negative findings can be attributed to under evaluation of gene-gene interactions and gene environment interactions [7] and/or because of missing some causative polymorphisms during linkage disequilibrium (LD) considerations [9]. *In silico* initiatives which take into account LD and compile tag SNPs and haplotypes can be very helpful in circumventing this.

Considering these aspects along with cumulative effect of multiple loci and complex disease heterogeneity, a fine tuning of the candidate gene approach has been sorted after [8,9]. Completion of the first phase of the 1000 genome sequencing project has further provided new avenues for reconsidering candidate gene association approaches to dissect the complexity of many genetic disorders. One major step in this regard could be a careful and thorough selection of candidate genes and variations forming the basis towards association-analysis. This support is available through various targeted *in silico* tools to evaluate all aspects of the candidate gene and the prioritised SNPs in a strategic manner.

This review intends to summarize current bioinformatics tools and literature available for the purpose of selecting a candidate gene for disease association studies and the genetic variants such as SNPs, from these candidate genes de novo, or from within a linkage peak. Some of these *in silico* methods are applicable to the functional analysis of data generated through various candidate gene association studies or for variants identified through post-GWAS fine mapping studies and/or next-generation sequencing.

### Selection of a candidate gene and retrieval of relevant sequence information

Recent data mining software advancements have catered substantially to growing research needs making it much easier to cope with the initial phase of searching through the enormous amounts of literature present online and keeping constantly updated in order to intelligently select a candidate gene. Tools provided by iHOP Web services (http://www.ihop-net.org/UniPub/iHOP/) [10] enables general literature mining and PubCrawler (http://pubcrawler.gen.tcd.ie/) [11] enables keeping track of daily updates. A commercial tool from Biovista - BioLab Experiment Assistant (http://www.biovista.com/bea/) [12] is uniquely designed around providing the user search capabilities to find concepts of interest (such as drugs, genes, molecules etc.) and review their interconnections visually, facilitating fine tuning of research strategy before getting down to reading literature. Ingenuity® Knowledge Base (http://www.ingenuity.com) is a depot of manually reviewed, enriched and sorted information of biological interactions and functional annotations, provided to the user through powered products like IPA®, iReport® in a contextual interface, also linking out to the original articles.

Analysing participating pathways is an important aspect of any gene’s functional analysis strategy. In this view, REACTOME (http://www.reactome.org) [13] is a cross referenced, manually curated and peer reviewed pathway database. LitInspector (http://www.litinspector.org) [14] and NetPath (http://www.netpath.org/index.html) [15] allow one to access curated signal transduction related literature and interaction pathways respectively. Predictive Networks (http://predictivenetworks.org/) [16] integrates gene interactions and networks information from PubMed literature and other online biological databases and presents it in an accessible and efficient user interface. Two other noteworthy commercial tools are GeneGo and Ingenuity IPA. GeneGo (http://www.genego.com/) technology facilitates pathway analysis to find interacting molecules and subsequent interactions relevant to the investigated trait or disease. Ingenuity IPA also considers pathway analysis in its package. Another website ToppGene suite (http://toppgene.cchmc.org/) [17], provides tools for functional enrichment of genes based on a training gene set (to be provided by user), and also for including protein networks and neighbouring genes of the locus in analysis.

Finding candidate genes for further investigation, also defined as gene prioritisation has been covered in detail elsewhere [18]. The hosted web portal – Gene Prioritization Portal (http://www.esat.kuleuven.be/gpp), links out to 33 current computational tools for this purpose, such as GeneRank, GeneWanderer, Caesar, SNPs3D and GeneDistiller among many others. This resource compares many online computational tools and thus, provides an efficient and comprehensive guide to help the user develop a suitable gene prioritisation strategy, and is highly recommended.

Functional analysis of a gene is incomplete without a brief investigation for any existing disease associations. OMIM®, Online Mendelian Inheritance in Man® (http://www.ncbi.nlm.nih.gov/omim) [19] is a database helpful to establish and/or investigate disease associations of gene of interest as it aims to lists all known genotype to phenotype correlations. PhenoPred (http://www.phenopred.org/) [20] is another useful starting resource for crosschecking for gene-disease association to set the stage and establishing a gene’s clinical relevance. An example of a disease specific web tool is Oncomine (https://www.oncomine.org) [21] providing an elaborate resource to cancer biologists interested in accessing cancer transcriptome data from large number of datasets collected, standardised and analysed as part of the Oncomine project. An elaborate description, comparison and usage strategy of tools currently available for the purpose of *in silico* gene function prediction relevant to cancer study, with their efficacy in suitably classifying uncharacterised cancer genes based on current knowledge from online databases has been given by Hu *et al.*[22].

Once a thorough assessment of literature and a holistic view of interacting pathways to the gene of interest have been considered, one is ready to focus on the gene composition and sequence. A gene locus can be analysed for various attributes. Many popular, consistently well updated and publically available databases provide genetic and functional information of a gene and its locus, which are advantageous to consider during SNP selection. Prior knowledge of the gene’s functional and structural elements within and those in its periphery can elicit a better understanding of the putative function of the gene variants. Comprehensive sites such as Entrez Gene (http://www.ncbi.nlm.nih.gov/Entrez) [23] and Ensembl (http://www.ensembl.org) [24] host an organised, collective resource linking out to various tools providing general information on gene structure, expression, splice variants encoded proteins, regulatory elements, SNPs and the like. Assessing splicing variants is of extreme importance when dealing with eukaryotic genomes, primarily due to their direct relation with candidate gene transcription, and also the acute sensitivity of splicing sites to SNP variations. Such an example has been elucidated in our recent study of the *Kallikrein15 (KLK15)* gene locus, where a SNP (rs266851) closely located (15 kb downstream) to a novel exon, renders increased susceptibility to ovarian cancer survival and is predicted to play a role in alternative mRNA splicing [25]. Another incidence is of a fairly common intronic *KLF6* gene polymorphism, called IVS1 -27 G > A, i.e. the *IVS*Δ*A* allele giving rise to an additional DNA binding site and increased expression of three alternative spliced transcripts of the gene [26]. Aceview (http://www.ncbi.nlm.nih.gov/IEB/Research/Acembly/) [27] provides an extensive annotated evaluation of cDNA supported transcriptome complete with data on mRNA and existing splice variants in the genome. The UCSC Genome Browser (http://genome.ucsc.edu/) [28] is an extremely efficient and popular tool, and extends to gauging genetic sequence information of gene loci in much detail. It provides, in numerous tracks, options to view transcript variants, repeats, evolutionary conservation and many other genetic modules which might be present in the gene of interest, and are relevant to the candidate SNPs under investigation. It also links to The Encyclopedia of DNA elements (ENCODE) (http://genome.ucsc.edu/ENCODE/) [29] which is a regularly updated database of functional and regulatory elements as found in the human and mouse genomes.

To assist in probing the functional importance of candidate genes while prioritising them, the VISTA Genome Browser (http://genome.lbl.gov/vista) [30] gives tools to compare your sequence with curated whole genome assemblies for regulatory elements and transcription factor binding sites (TFBS). It also links to VISTA Enhancer browser (http://enhancer.lbl.gov/) [31], which is a database of experimentally validated human enhancer elements, and to VISTA Region viewer (http://rviewer.lbl.gov/) [32], a tool for prioritising genomic regions for further studies. Another recommended tool is VarioWatch (Previously GenoWatch) (http://genepipe.ncgm.sinica.edu.tw/variowatch/main.do) [33] which retrieves comprehensive gene information in a particular region, in real time from various primary sources.

Various high-end computational resources developed in the last decade are freely available online and are updated continuously, although some limitations exist. Tools which rely on Gene Ontology (GO) to characterise information are limited because the GO annotation is an ongoing process, and cannot provide a full picture. Also, it shows a bias towards well known, better characterised diseases and research terms, hence, making the search miss on what could otherwise be functionally relevant to the gene under investigation [34]. Thus tools which support descriptive keyword search to identify desired genes are more useful in some cases. Care should be taken to use the most updated versions of tools available online, as these have been fine tuned to have better accuracy rates, are also backed by latest database resources such as the 1000 genomes project (in relation to genomic variants) and the latest genome assembly which is currently GRCh37/hg19 which can be tracked for updates at the Genome Reference Consortium website (http://www.ncbi.nlm.nih.gov/projects/genome/assembly/grc/).

A consolidated account of all the above mentioned resources is provided in Additional file 1: Table S1.

#### Cataloguing SNPs in a candidate gene

Once the relevance of the candidate gene and the spread of its functional elements (enhancer, promoter, intron, exon, UTR etc.) have been noted, the next step is cataloguing the SNPs present in the candidate gene, in its regulatory sequences (Promoter, TF binding sites, non coding regions), and in its surrounding regions which might have long distance effects on the gene function (eg. enhancers). A demonstration of this step and its context can be found in a recent review by us focussed on cataloguing all SNPs important to the Kallikrein gene locus [35].

Some resources which facilitate scouring the gene locus for submitted variants have been reviewed extensively by Coassin *et al.*[36] such as NCBI’s dbSNP (http://www.ncbi.nlm.nih.gov/snp) [37,38]. PolyScan (http://genome.wustl.edu/pub/software/polyscan/) [39] can be used to reprocess the results to improve detection. BioQ (http://bioq.saclab.net/) [40] enables one to track back to the experimental process flow and data source of the variant data. dbSNP-Q (https://cgsmd.isi.edu/dbsnpq/) [41] provides a downloadable interface which can be used to meaningfully analyse dbSNP data with custom designed tables which use task based queries to select and display relevant information. SNPper (http://snpper.chip.org/) [42], one of the tools provided at the CHIP bioinformatic tools website enables retrieval of SNP based on name or gene association and compliments it with additional useful tools such as FlankXtender to include sites flanking the gene. Evaluating functional elements in the genome for putative variations can be performed by RAVEN (Regulatory Analysis of Variation in ENhancers) (http://www.cisreg.ca/) although the link is not currently functional [43]. This is a web application specially designed to identify genetic variations in cis regulatory elements of the candidate gene through combining consideration of transcription, TFBS prediction and phylogenetic footprinting, enabling researchers to isolate SNPs which might have a direct consequence on transcriptional regulation of the genomic site [43]. A database providing sequenced and genotyped SNPs in genes implicated in cancer studies is the SNP500Cancer (http://variantgps.nci.nih.gov/cgfseq/pages/snp500.do) [44] hosted by the Variant GPS (http://variantgps.nci.nih.gov/cgfseq/pages/home.do). ANNOVAR (http://www.openbioinformatics.org/annovar/) [45] enables mining through the data from high throughput experiments and identifying, sorting, and prioritising candidate SNPs (variants) in important genomic regions in its filter based annotation. The SNPinfo Web Server (http://snpinfo.niehs.nih.gov/) [46] provides many efficient, comprehensive and user friendly tools suited for various purposes such as GenePipe (for Candidate gene selection), GenomePipe (Functional SNP selection), LinkagePipe (SNP selection in one genomic loci of interest), TagSNP, FuncPred (querying SNP function prediction) and SNPseq (viewing SNPs in their genomic region context, with information on CpG sites), making this a one stop website for initial SNP investigation from scratch. All mentioned tools, which can be used for SNP cataloguing, are detailed in Additional file 1: Table S1; which also shows schematically the tools available and places the important step of choosing an SNP of interest in the context of candidate gene association studies.

### Selection of the tag SNPs for the association studies

Linkage disequilibrium is a phenomenon where alleles associate at different loci non-randomly; carrying with them conserved combinations of SNPs. The most widely recognised measure for LD is *r*^*2*^, where r is the correlation coefficient between two loci with alleles in association [47]. A gene locus hosting SNPs demonstrating LD have a higher propensity to be conserved in populations with recombination occurring on either side of it [48]. Analysis of LD within the candidate SNPs is a valued way of narrowing down on the limits of the disease susceptible genomic region [48]; because they will mostly be inherited together and show similar frequencies in affected individuals of a population. Such SNPs closely linked with each other and demonstrating LD effects can be tagged and represented by selected SNPs among them, referred to as tag SNPs. LD and its evolutionary and medical importance has been described in detail in several reviews [48,49]. Recently developed resources specific to LD analysis are, DistiLD http://distild.jensenlab.org/[50], GLIDERS (http://www.sanger.ac.uk/resources/software/gliders/) [51], SNPAnalyser 2.0 (http://snp.istech21.com/snpanalyzer/2.0/) [52] further elaborated in Additional file 1: Table S1. SNAP (SNP Annotation and Proxy Search; http://www.broad.mit.edu/mpg/snap/) [53] further includes data from the 1000 genomes project in its data pool and also provides graphical representations of regional LD analysis. A web link to multiple LD tools can be found at http://www.genes.org.uk/software/LD-software.shtml[54].

Haplotypes can be defined as evolutionary conserved segments of DNA inherited together. It is at these regions that tag SNPs and LD effects are observed; such that genotyping one SNP in a locus can determine the effects of many others. The international HapMap project (http://hapmap.ncbi.nlm.nih.gov/) took the initiative of genotyping sections of human populations worldwide to bring the haplotype map, and accelerate the search for Haplotypes and tag SNPs to narrow down on statistically significant, reviewed disease associated loci, while understanding the patterns of genetic distribution in humans from diverse regions [5]. It currently provides this data to allow further analysis and interpretation of GWAS results with the use of imputation. A resource like Haploview (http://www.broad.mit.edu/mpg/haploview/) [55] takes its data resource from HapMap project and can assist greatly in LD analysis during gene and SNP prioritisation.

Following the identification of candidate SNPs and a peripheral analysis of their location in the genome, focussed computational tools designed to specifically understand downstream effects of SNPs depending on their genomic context and placement can be considered, thus, enabling in depth *in silico* analysis of the respective functional changes they might bring in cellular processes.

### Selection of candidate SNPs through function prediction

SNPs are classified according to their location in the gene locus, which also most times dictates the functional downstream effects of the SNP [56] and will guide the selection of appropriate computational tools towards its analysis. SNPs within the coding region of the gene but not causing any change in the formed protein, such that both alleles still encode the same protein sequence, are classified as synonymous SNPs. This is possible due to the degeneracy of the genetic code; and it does not cause any direct functional defects than from probable splicing variations. SNPs in the coding region which leads to a change in the translated amino acids and thus in the encoded protein are categorised as non-synonymous SNPs (nsSNPs), as encoded protein sequences differ between both alleles. While the functional role of non-synonymous SNPs is relatively straight forward, SNPs located in regulatory and intronic regions have recently gained importance upon recognition of their potential to deregulate transcriptional efficiency, gene expression and splicing [57-60]. Especially SNPs in regions encoding microRNA and non-coding RNAs can thus be considered for association studies [61]. An interesting tool to use in the start is the Variant Effect Predictor (http://www.ensembl.org/info/docs/variation/vep/index.html) [62] found within the Ensemble periphery which predicts the functional effect of known and unknown variants. Given below are detailed web tools specific to analyse SNPs in coding regions and in regulatory regions.

### Functional SNPs within the coding regions

A nsSNP affect protein sequence and structure, and can affect its functionality depending on the position of the change and the amino acid it replaces. Usually such changes differ in the degree of deleterious effect they cause, with highly deleterious SNPs already being filtered out by nature through natural selection [63]. Thus, all nsSNPs discovered through high throughput studies, can be those with long ranging clinical implications to disease causation, and even though occurring in low frequency, are none the less quite important. For example, rs17632542 in the *KLK3* gene is implicated in high association to prostate cancer susceptibility, and rs1126497 with a C/T polymorphism in epithelial cell adhesion molecule (*EpCAM*) in its exon 3 has been linked to increased risk of breast cancer in chinese populations [64]. The knowledge of gain or loss of function attributed to a protein by the incidence of a nsSNP can be acquired by further functional analysis and experimental analysis. Analysis can begin with annotating the resulting protein sequence and structure of the variant carrying the SNP. Then subsequent investigation into functional aspects such as its conformation, enzymatic sites and amino acid interactions will reveal how the variation affects protein structure and function of the resultant phenotype. Additional file 2: Table S2 lists useful resources in this area.

Annotation of protein structure can be performed by resources such as SNPs3D (http://www.snps3d.org/) [65]. LS-SNP/PDB (http://ls-snp.icm.jhu.edu/ls-snp-pdb/) [66] lets one map the variations on 3D structures available in Protein Data Bank. ModBase (http://modbase.compbio.ucsf.edu) [67] goes one step further allowing comparative annotated protein structure models, also linking out to functional analysis of the SNP effect on protein. Data from various algorithms and functional criteria applied to the dbSNP dataset have been integrated by PolyDoms (http://polydoms.cchmc.org) [68] to predict structural and functional protein variations, also integrating data on pathways, interactions and allelic variations from various sources [34]. UniProt (http://www.uniprot.org/) [69] provides a database for protein information while the direct SNP effect on protein function can be studied using SNPeffect (http://snpeffect.switchlab.org/) [70], and Pupasuite (http://pupasuite.bioinfo.cipf.es/) [71]. These, apart from providing other tools as discussed later, annotate protein structures and facilitate checking the protein for functional sites such as catalytic sites, DNA and protein binding sites and also those harbouring post translational modifications ([34] and references therein). Users should note that knowing the methodology used by the software is imperative to assess the accuracy and its relevance to the case being investigated [36].

Recent advancements in forecasting the effects of amino acid substitutions in protein sequence train computational tools to learn and then predict downstream effects of protein variants. These programs are trained by using either disease-associated alleles in databases or by experimentally varying amino acid substitutions to check for functional changes [34]. Many recent tools have been described and compared in the review by Mah *et al*. [72], albeit in a different mode of classification. Mah *et al.*[73] classify available algorithms as sequence versus structure based approaches, detailing advantage and drawbacks to both. The sequence-based approach uses induction of single base substitutions to predict effects on the function of resulting proteins [74], for example as PoPMuSiC (http://babylone.ulb.ac.be/popmusic/) [75] checks for structural stability; Mutation Profiling (http://profile.mutdb.org/) [76] predicts effects of amino acid substitutions, whereas, the structure-based approach elucidates the altered phenotype caused by the protein by predicting effects on its 3D structure [72,77], and its major drawback is restrictive data availability as structural information is not yet available for many proteins [72]. PolyPhen-2 (http://genetics.bwh.harvard.edu/pph2/) [78] and SIFT (http://sift.jcvi.org/) [79] are two sequence based resources for predicting the functional effect of human SNPs under investigation. Polyphen is less dependent on the multiple alignments used as input. If user alignments for a specific dataset are not available for input, then Polyphen could perhaps be preferred for this reason. On the other hand, if own alignments can be produced then SIFT might be preferable since its web interface allows one to specify the alignment. PROVEAN (http://provean.jcvi.org/) [80] is a tool which also takes in frame insertions, deletions and multiple amino acid substitutions into consideration, being more relevant to variation analysis from next generation sequencing projects.

Lately, variants affecting the post translational protein modifications have received attention, in their potential role in disease causation. Post translational modifications can be reversible or irreversible changes made to a protein after its translation, changing its function by changing protein structure and dynamics or by altering a binding site on it, thus playing an important role in signal transduction pathways and modulating a protein’s cellular role [81]. Close to 200 post translational modifications have been recently discovered in the human cell, most of which either facilitate binding of a chemical group to a protein or proteolytic cleavage of the protein [82]. When a polymorphism occurs in a post-translational target site, it can invariably result in a host of downstream effects causing disease or its susceptibility. NetPhos (http://www.cbs.dtu.dk/services/NetPhos/) [83] is a tool which uses artificial neural networks to predict phosphorylation sites in submitted input sequences, determining susceptible regions and facilitating further checks for disease causation. A resource like PROSITE (http://prosite.expasy.org/) [84] can be used to predict the occurrence of these target sites in and near the SNP, and can be used to analyse the functional repercussion of the polymorphism proximity to the motif.

Keeping the wide range of available products in mind, protein prediction tools should be assessed for the method they follow to determine protein structure variations and the functional causal effect. This can be done by reading their respective manuscripts in detail, and to determine if that method suits, and is the best one for the investigation.

### Functional SNPs within the non-coding and regulatory regions

Eukaryotic gene expression involves multiple steps: gene transcription, processing of RNA through splicing mechanisms, translation into a protein product, post-translational modifications and subsequent protein activity. The majority of gene expression regulation takes place within genetic elements modulating it, like enhancers and silencers, TFBS and splicing sites. The intricacies with which sequence variation give rise to gene expression defects have been covered by Wang *et al.*[58]. Many computational tools have been developed to aid investigation of SNP effects in each of the above stages of gene expression regulation.

a) SNPs in regulatory elements

SNPs within the regulatory elements of the gene can disrupt gene expression by altering TFBS, influencing the strength of enhancers and promoters, making these SNPs of prime importance to be considered for candidate gene association studies [84]. Below, we list tools for general investigation of genomic region for regulatory elements, in order to filter the genomic regions hosting functional SNPs; and then we move to list tools specifically investigating predicted influence of candidate SNPs on any such region.

Putative genetic regulatory elements such as promoter regions, TFBS, CpG islands over-seeing gene expression, along with microRNA binding sites, are extremely crucial locations where a SNP can cause widespread expression variations and potential disease causing effects, perhaps in a tissue specific nature. Examples of such genetic alterations are discussed by Werner *et al.*[85]. Information on the spread of these regulatory modules can be obtained from previously mentioned regular sequence databases and software like UCSC genome browser, and Pupasuite [71]. An initial DNAase hypersensitivity check from ENCODE (if available for cell type) using the UCSC genome browser can show open and closed chromatin regions to verify the epigenetic context of the locus. Open chromatin regions indicate exposed regulatory sites hosting important functional elements like transcription factors binding sites, enhancers and ncRNAs [34,86]. Such regions which can be very important in de-differentiation diseases like cancer where epigenetic aberrations are frequent and could have a potential causative nature [87].

Analysis of the regulatory regions involves starting at a few well known websites to find TFBS such as TFBIND (http://tfbind.hgc.jp/) [88], MatInspector (http://www.genomatix.de/matinspector.html) [89], TFSEARCH (http://www.cbrc.jp/research/db/TFSEARCH.html) [90], MAPPER (http://bio.chip.org/mapper) [91] and also is-rSNP (http://www.genomics.csse.unimelb.edu.au/product-is-rSNP.php) [92] and RegulomeDB (http://www.regulomedb.org/index) [93], which scans SNP sites for significant potential regulatory elements such as transcription factor binding and histone modifications. FunciSNP, a recent tool available at http://bioconductor.org/ which is itself a rich source of multi-purpose bioinformatic tools, takes into account chromatin features along with tag SNP and linked SNPs from the 1000 genomes project to spew out functionally important SNPs specific to non-coding regions [94]. A rare variant rs183373024, was recently explored using FunciSNP and implicated in prostate cancer risk based on its position in a transcription factor occupied region; disrupting a FoxA1 binding site at 8q24 [95].

In certain hormone mediated diseases such as cancer, hormone response elements have causal relations with aberrant hormonal modulations [96]. Thus, promoter regions of candidate genes can be analysed by tools like Dragon ERE Finder (http://datam.i2r.a-star.edu.sg/ereV3/index.html) [97] and JASPER (http://jaspar.genereg.net/) [98] to characterise for the presence of putative estrogen and androgen response elements (EREs and AREs) respectively. CISTER (http://zlab.bu.edu/~mfrith/cister.shtml) [99] can be used to check for both elements. These tools and strategy were used by Batra *et al.*[25] for a similar purpose.

A recent class of powerful functional elements, which play an extensive role in the genomic regulation as a part of epigenetic mechanisms in the cell, are microRNAs [100]. Their deregulation has been implicated in various diseases like Cancer, Schizophrenia and Autism [101-103]. SNPs lying in miRNA binding regions and interfering with its regulatory function, also called MiRSNPs have also been reported to be associated with risk and with drug resistance in some instances [101]. mirBase (http://www.mirbase.org/) [104] is a microRNA Database which finds targets predicted by microCosm, TargetScan and Pictar [105]. Mirsnpscore (http://www.bigr.medisin.ntnu.no/mirsnpscore/) [106] is a database of SNPs predicted to influence microRNA efficacy by mapping potential causative SNPs to microRNA target sites. MirSNP (http://cmbi.bjmu.edu.cn/mirsnp) [107] provides a database of SNPs which are predicted to enhance/create or decrease/break a miRNA-mRNA binding site. Another tool to find microRNA targets is microRNA.org (http://www.microrna.org/) [108], which also provides experimentally observed gene expression patterns. Two available and well recommended resources for miRSNP information and functional effect prediction in diseases are PolymiRTS database (http://compbio.uthsc.edu/miRSNP/) [109] and Patrocles (http://www.patrocles.org/) [110]. A comparative strategy of using more than one web tool can increase scope of analysis and circumvent technical drawbacks of the individual tools.

b) eQTL

Expression quantitative trait loci (eQTL) mapping is a technique which uses results from two high throughput techniques, i.e. genome wide gene expression analysis and the GWAS to define an association between a particular genomic loci variant with a changed gene expression pattern, thus, attributing specific genetic regulatory roles to candidate SNPs in the gene locus [111-114]. *cis* acting eQTLs are those located near the target genes and have a direct influence on its gene regulation, whereas *trans* acting eQTLs are located away from the target region and show an indirect remotely regulated gene expression [115].

Software developed towards facilitating mining of genetic expression and variant associations include eQTL Explorer, eQTL Viewer, FastMap and Lirnet. Bioinformatics concepts relating to eQTL have been reviewed in [116]. eQTL Explorer (http://web.bioinformatics.ic.ac.uk/eqtlexplorer/) [117] as an addition to resources provided by previous softwares like WebQTL [118] and QTL Express [119], enables integrated visualization using a Java graphical interfaces; extracts eQTL results from external sources (multiple microarray experiments) and presents them such that they can be compared among each other, and with the pQTL (protein expression) mapped to the genome. eQTL Viewer (http://statgen.ncsu.edu/eQTLViewer/) [120] uses Scalable Vector Graphics for visualisation and carries an added advantage of biological annotations being present dynamically on its interactive mapping results plot. FastMap (http://comptox.unc.edu/fastmap.php) [121], developed in 2009, enables a faster analysis of expression and genotype data by organising SNPs into a hamming distance based tree thus minimizing the number of steps involved. In addition, it provides permutation based significance testing of results. Lirnet (http://www.cs.washington.edu/homes/suinlee/lirnet/) [122] uses a learning strategy to overcome problems of low population size and correlating SNP effect on gene expression due to large genomic regions being in LD for any given trait, i.e. it learns the ‘regulatory potential’ of a SNP through a Bayesian method from its previously known genomic context (such as regulatory networks and features existing and relevant to the gene) and gives an estimate of likelihood of effecting gene expression.

## Conclusion

Recent advances in high-throughput experimental technologies like whole-genome gene expression profiling, the genome wide association studies (GWAS), next generation DNA, RNA sequencing and CHIP-seq scan the genome for disease associated genetic variants and add knowledge to gene function, regulation, SNP prioritisation resources [123,124]. They provide extensive whole genome data and high coverage genomic, transcriptomic, epigenomic, and proteomic information in numerous cell types, classifying tissue specific behaviour, interactions and cell functioning [124,125]. In present day context, candidate gene studies can utilize the current knowledge resources made available by these initiatives to further discovery, and validating these interactions to uncover a myriad of susceptible disease associations. Applying the candidate gene approach to next generation data is bound to give rich dividends in terms of elucidation of complex disease mechanisms, better prognosis and diagnosis of patients in a short time, and in an efficient way.

## Competing interests

The authors declare that they have no competing interests.

## Authors’ contributions

JB conceived the idea. RP collated the information on all in silico tools and associated websites with input from JB. RP and JB wrote the manuscript. JAC critically reviewed the manuscript. All authors read and approved the final manuscript.

## Supplementary Material

Additional file 1: Table S1 List of useful web-tools for candidate gene selection and SNP mapping [5,6,10-21,23,24,27-33,37,39-46],[50-55,62]. Click here for file

Additional file 2: Table S2 SNP Effects and functional analysis [28,62,65-71,75,76,78-80,83,84],[88-94,97-99,105-110,117,120-122,126-139]. Click here for file
