# Supplementary material for: Candidate gene association studies: a comprehensive guide to useful in silico tools
Source: BMC Genet. 2013 May 9;14:39. doi: 10.1186/1471-2156-14-39 (PMC3655892; doi:10.1186/1471-2156-14-39)
Supplement: Additional file 1: Table S1 — List of useful web-tools for candidate gene selection and SNP mapping [5,6,10-21,23,24,27-33,37,39-46],[50-55,62]. [file 1471-2156-14-39-S1.doc]

**Additional file 1: Table S1: List of useful web-tools for candidate gene selection and SNP mapping**

| **Name** | **Website** | **References** | **Comments** | **Special feature** | **Interface /Platform** |
| --- | --- | --- | --- | --- | --- |
| LITERATURE MINING | | | | | |
| **iHOP (Information hyperlinked over proteins) Web services** | <http://www.ihop-net.org/UniPub/iHOP/> |  | Extensive Literature mining –publically accessible, Source: Pubmed | Provides experimental interaction network. Organise found information through “gene model” building. | Graphic User Interface/Web Based |
| **PubCrawler** | <http://pubcrawler.gen.tcd.ie/> |  |  | Tracks and informs about daily updates on PubMed and GenBank |  |
| **Biovista - BioLab Experiment Assistant** | <http://www.biovista.com/bea/> |  | Commercial | Multipurpose Literature and conceptual review with data mining |  |
| **GeneGo** | <http://www.genego.com/> |  | Commercial | Multipurpose Literature and conceptual review with data mining |  |
| **Ingenuity® Knowledge Base** | <http://www.ingenuity.com/products/ipa> |  | Commercial | Multipurpose Literature and conceptual review with data mining |  |
| **REACTOME** | [http://www.reactome.org](http://www.reactome.org/) |  | Cross referenced, manually curated and peer reviewed pathway database | Gives tools to browse reactions, pathway information of biological processes, and also to analyse gene expression data |  |
| **LitInspector** | [http://www.litinspector.org](http://www.litinspector.org/) |  | Manually curated synonyms based Literature mining | Colour coded user interface for word search according to Gene, Transcription factor, Disease, Pathway per Journal and abstract |  |
| **NetPath** | <http://www.netpath.org/index.html> |  | Expert curated resource for signal transduction; NetSlim provides curated Pathway maps | Currently contains 10 immune and 10 cancer signalling pathways with gene interactions and corresponding experiments |  |
| **Predictive Networks** | <http://predictivenetworks.org/> |  | Enables prediction of potential gene function by consolidating experimental and theoretical gene data | Pathway Analysis and literature mining of gene and its interactants |  |
| CANDIDATE GENE PRIORITIZATION | | | | | |
| ***Web tools*** | | | | | |
| **ToppGene suite** | <http://toppgene.cchmc.org/> |  | The requirement for training gene sets can be a drawback | A set of ToppFun, ToppGene, ToppNet, ToppGenet analytic tools | Graphic User Interface/Web Based |
| **Gene Prioritization Portal** | <http://www.esat.kuleuven.be/gpp> |  | Highly recommended | Comprehensive Guide to 33 computational tools for Candidate Gene prioritisation, including strategy. |  |
| ***General Information*** | | | | | |
| **OMIM**®**, Online Mendelian Inheritance in Man** **®** | <http://www.ncbi.nlm.nih.gov/omim> |  |  | Database of annotated genotype to phenotype associations | Graphic User Interface/Web Based |
| **PhenoPred** | <http://www.phenopred.org/> |  |  | Gives gene-disease associations and also predicted genes in a disease |  |
| **Oncomine** | [https://www.oncomine.org](https://www.oncomine.org/) |  | Cancer related | Contains cancer transcriptome data from 699 samples (accessed March 2013) |  |
| **Entrez Gene** | <http://www.ncbi.nlm.nih.gov/Entrez> |  | Comprehensive resources;  updated frequently; Link out to other Databases/Web links for follow up study | Highly recommended; cross connected sister tools provide flexibility in use |  |
| **Ensembl** | [http://www.ensembl.org](http://www.ensembl.org/) |  |  | Extensive analytical tools for human, Mouse and Zebra fish genomes; Provides Variant Effect Predictor |  |
| **Aceview** | [www.ncbi.nlm.nih.gov/IEB/Research/Acembly/](http://www.ncbi.nlm.nih.gov/IEB/Research/Acembly/) |  |  | Extensive annotated transcriptome resource |  |
| **UCSC Genome Browser** | <http://genome.ucsc.edu/> |  |  | Customisable tracks for graphical representation of genomic region; Table browser option and other intrinsic utilities |  |
| **The Encyclopedia of DNA elements (ENCODE)** | <http://genome.ucsc.edu/ENCODE/> |  |  | Comprehensive resource for functional elements of select genomic data |  |
| **VISTA Genome Browser** | <http://genome.lbl.gov/vista> |  |  | Comparative analytical tools or genomic sequences (user’s or predefined) from different species |  |
| **VISTA Region viewer** | <http://rviewer.lbl.gov/> |  |  | Genomic interval prioritization for functional studies |  |
| **VarioWatch (Previously GenoWatch)** | <http://genepipe.ncgm.sinica.edu.tw/variowatch/main.do> |  | Gene mining/SNP Mining | Easy to use interface with options to read known ontology of every gene/SNP in its functional context |  |
| ***Gene functional analysis*** | | | | | |
| **VISTA Genome Browser** | <http://genome.lbl.gov/vista> |  | Link out to other Databases/Web links for follow up study | Comparative analytical tools or genomic sequences (user’s or predefined) from different species | Graphic User Interface/Web Based |
| **VISTA Enhancer browser** | <http://enhancer.lbl.gov/> |  |  | Whole genome enhancer browser. Information on 991 elements (as seen on Feb2013) gene enhancer activity |  |
| SNP PRIORITIZATION | | | | | |
| ***SNP cataloguing*** | | | | | |
| **1000 genomes project consortium** | <http://www.1000genomes.org/> |  | Provides control data listing variants at frequency of >=1% in the population. | Whole genome sequence data for 2500 individuals among different ethnic backgrounds. | Raw data and Graphic user interface/Web based |
|  | 1000 genomes phase 1 variants can be accessed at <http://browser.1000genomes.org/> |  |  |  |  |
|  | All data can be accessed at mirrored sites EBI |  |  |  |  |
|  | FTP: <ftp://ftp.1000genomes.ebi.ac.uk/vol1/ftp/> |  |  |  |  |
|  | NCBI FTP: <ftp://ftp-trace.ncbi.nih.gov/1000genomes/ftp/> |  |  |  |  |
| **VarioWatch (Previously GenoWatch)** | <http://genepipe.ncgm.sinica.edu.tw/variowatch/main.do> |  | SNP Mining | Easy to use interface with options to read known ontology of every gene/SNP in its functional context | Graphic user interface/Web based |
| **NCBI’s dbSNP** | <http://www.ncbi.nlm.nih.gov/snp> |  | Contains information on SNPs, Insertions, deletions and tandem repeats, microsatellites | Provides sequence context of said variants with their frequency of occurrence and details on experiment | Raw data resource; Graphic user interface/Web based |
| **PolyScan** | <http://genome.wustl.edu/pub/software/polyscan/> |  |  | . | Graphic user interface/Web based |
| **BioQ** | <http://bioq.saclab.net/> |  | Database information | Back tracks experimental process flow and data source |  |
| **dbSNP-Q** | <https://cgsmd.isi.edu/dbsnpq/> |  | Downloadable interface | Analyse dbSNP data with custom designed tables which use task based queries |  |
| **SNPper** | <http://snpper.chip.org/> |  | Provides additional tools | Retrieval and analysis of human SNPs |  |
| **RAVEN (Regulatory Analysis of Variation in ENhancers)** | <http://www.cisreg.ca/> |  | Non-functional link | Analyses regulatory genomic regions eg. Enhancers for underlying variants |  |
| **Ingenuity® Variant analysis** | <http://www.ingenuity.com/products/ingenuity_variant_analysis.html> |  | Commercial | Comprehensive; Strategically identifies casual variants; enables additional scoping for relevance (considering biological interactions, established disease genes, role in signalling pathways including drug interactions) |  |
| **SNP500Cancer** | <http://variantgps.nci.nih.gov/cgfseq/pages/snp500.do> |  | Disease specific | Database for SNPs implicated in genes functionally important in Cancer |  |
| **ANNOVAR** | <http://www.openbioinformatics.org/annovar/> |  | Recommended | Extensive scope of functionality analysis; gene based, region based, filter based and other | Perl interface; For Windows users prior installation if MSYS and CYGWIN are recommended for ensuring functionality |
| **SNPinfo Web Server** | <http://snpinfo.niehs.nih.gov/> |  | Highly recommended | A collection of efficient tools in one web site, covering all aspects of gene prioritisation. | Graphic user interface/Web based |
| **Variome** | [http://variome.net](http://variome.net/index.php/Main_Page) |  | Recommended | A Web Resource with a variety of SNP analysing tools for functional annotation of SNP |  |
| **Variant Effect Predictor** | <http://www.ensembl.org/info/docs/variation/vep/index.html> |  | Previously known as SNP Effect predictor, Missense SNPs prediction link out to SIFT and PolyPhen | Predicting function of known and unknown SNPs | Available as Web interface/ perl script/ Ensemble’s perl API |
| ***Selection of Tag SNPs for the association studies*** | | | | | |
| **The International HapMap Project** | <http://hapmap.ncbi.nlm.nih.gov/> |  | Comprehensive resources;  updated frequently; Link out to other Databases/Web links  for follow up study | Aims to collect haplotypes among African, Asian, and European ancestry such that researchers can analyse tag SNPs and link candidate gene/variants to disease susceptibility | Graphic user interface/Web based |
| **DistiLD** | <http://distild.jensenlab.org/> |  | Broad representation of genetic variant and its effect | Considers SNP’s as LD blocks and represents them in the simple context of chromosome, gene and disease. |  |
| **GLIDERS ( Genome-wide LInkage DisEquilibrium Repository and Search engine)** | <http://www.sanger.ac.uk/resources/software/gliders/> |  | Database using HapMap phase 2 and 3 data | Can investigate long ranging LDs (>500 kb) and short too |  |
| **SNPAnalyser 2.0** | <http://snp.istech21.com/snpanalyzer/2.0/> |  | Software | Can handle large data sets in a short time |  |
| **SNAP (SNP Annotation and Proxy Search)** | <http://www.broad.mit.edu/mpg/snap/> |  | **Data resource** | **Comprehensive** |  |
|  |  |  | 1000 genomes project (<http://www.1000genomes.org/>), The international HapMap Project ( <http://hapmap.ncbi.nlm.nih.gov/>) |  |  |
| **A Compilation of Some Available Software for Linkage Disequilibrium Analysis** | <http://www.genes.org.uk/software/LD-software.shtml> |  |  |  | Web Link |
| **Haploview** | [www.broad.mit.edu/mpg/**haploview**/](http://www.broad.mit.edu/mpg/haploview/) |  | **Data resource**  The international | **Comprehensive** | Graphic user interface/Web based |
|  |  |  | HapMap Project ( <http://hapmap.ncbi.nlm.nih.gov/>) |  |  |
