# Supplementary material for: Candidate gene association studies: a comprehensive guide to useful in silico tools
Source: BMC Genet. 2013 May 9;14:39. doi: 10.1186/1471-2156-14-39 (PMC3655892; doi:10.1186/1471-2156-14-39)
Supplement: Additional file 2: Table S2 — SNP Effects and functional analysis [28,62,65-71,75,76,78-80,83,84],[88-94,97-99,105-110,117,120-122,126-139]. [file 1471-2156-14-39-S2.doc]

**Additional file 2: Table S2 SNP Effects and functional analysis**

| **Name** | **Website** | **References** | **Comments** | **Unique aspect of the tool** | **Interface /Platform** |
| --- | --- | --- | --- | --- | --- |
| SNP IN CODING REGION - NON SYNONYMOUS SNPS | | | | | |
| ***Changes in Protein function*** | | | | | |
| **Variant Effect Predictor** | <http://www.ensembl.org/info/docs/variation/vep/index.html> |  | Previously known as SNP Effect predictor, Missense SNPs prediction link out to SIFT and PolyPhen | Predicting function of known and unknown SNPs | Available as Web interface/ perl script/ Ensemble’s perl API |
| **SNPs3D** | <http://www.snps3d.org/> |  | Easy user interface; provides web links to follow up analysis | Predicts effects of non synonymous SNPs in an input gene with respect to stability of protein | Graphic user interface/Web based |
| **LS-SNP/PDB** | <http://ls-snp.icm.jhu.edu/ls-snp-pdb/> |  |  | Lets one map the variations on 3D structures available in Protein Data Bank. |  |
| **ModBase** | [http://modbase.compbio.ucsf.edu](http://modbase.compbio.ucsf.edu/) |  | Comparative annotated protein structure models | Predicts the structure of the protein model and compares between species and various available transcript variants; Other follow up tools accessible at the Web link |  |
| **PolyDoms** | [http://polydoms.cchmc.org](http://polydoms.cchmc.org/) |  | Recommended | Integrates data on pathways, interactions and allelic variations during functional and structural prediction of SNP effect Has filters to choose type of SNP. | Graphic user interface/Web based |
| **UniProt** | <http://www.uniprot.org/> |  | Basic comprehensive database for protein sequence and functional information |  |  |
| **SNPeffect** | <http://snpeffect.switchlab.org/> |  | Comprehensive functional analysis and annotation of variants | Detailed variant analysis of disease and human proteome with the following tools: TANGO, WALTZ, LIMBO and FOLDx |  |
| **Pupasuite** | <http://pupasuite.bioinfo.cipf.es/> |  | Might take processing time | Provides filters for checking SNPs in regulatory elements |  |
| **PoPMuSiC** | <http://babylone.ulb.ac.be/popmusic/> |  |  | Tests single site mutations in proteins (*in silico)* to check for changes/effect in stability of protein structure |  |
| **Mutation Profiling** | <http://profile.mutdb.org/> |  |  | Functional Analysis and prediction of loss or gain of function by Amino Acid Substitutions |  |
| **PolyPhen-2** | <http://genetics.bwh.harvard.edu/pph2/> |  |  | Functionality analysis of amino acid substitutions |  |
| **SIFT** | <http://sift.jcvi.org/> |  |  | Functionality analysis of amino acid substitutions |  |
| **PROVEAN** | <http://provean.jcvi.org/> |  |  | Functionality analysis of single, multiple amino acid substitutions, insertions and deletions | Graphic user interface/Web based |
| ***Post translational Modifications*** | | | | | |
| **NetPhos** | <http://www.cbs.dtu.dk/services/NetPhos/> |  | Characterises phosphorylation sites on protein sequence |  | Graphic user interface/Web based |
| **PROSITE** | <http://prosite.expasy.org/> |  | Functional annotation of protein sequence and motifs | Checks for functional effects of SNP near a posttranslational modification motif | Graphic user interface/Web based |
| SNP IN NON-CODING REGION | | | | | |
| ***Changes in Gene Regulation*** | | | | | |
| *Changes in Transcription Factors and Promoter regions* | | | | | |
| **UCSC Genome Browser** | <http://genome.ucsc.edu/> |  |  |  | Graphic user interface/Web based |
| **Pupasuite** | <http://pupasuite.bioinfo.cipf.es/> |  |  |  |  |
| **Genetic Variation Database** | <http://www.humgen.nl/SNP_databases.html> |  |  | A extensive collection of listed links to tools for various operations concerning SNP analysis | Web Link |
| **TFBIND** | <http://tfbind.hgc.jp/> |  |  |  | Graphic user interface/Web based |
| **MatInspector** | <http://www.genomatix.de/matinspector.html> |  |  |  |  |
| **TFSEARCH** | <http://www.cbrc.jp/research/db/TFSEARCH.html> |  |  |  |  |
| **MAPPER** | <http://bio.chip.org/mapper> |  |  |  |  |
| **is-rSNP** | <http://www.genomics.csse.unimelb.edu.au/product-is-rSNP.php> |  | Recommended |  |  |
| **RegulomeDB** | <http://www.regulomedb.org/index> |  | Recent | Scans SNP sites for significant potential regulatory function and elements | Graphic user interface/Web based |
| **FunciSNP** | <http://bioconductor.org/> |  | Recent | Takes into account chromatin features during functional prioritization of SNP |  |
| **Dragon ERE Finder** | <http://datam.i2r.a-star.edu.sg/ereV3/index.html> |  | Useful for investigating hormone mediated diseases | Finds Estrogen response elements (ERE) | Graphic user interface/Web based |
| **JASPER** | <http://jaspar.genereg.net/> |  |  | Finds Androgen response elements (ARE) along with various other transcription factor binding profiles |  |
| **CISTOR** | <http://zlab.bu.edu/~mfrith/cister.shtml> |  |  | Finds Estrogen response elements (ERE) and Androgen response element (ARE); and other cis-acting regulatory elements |  |
| *Changes in microRNA induced regulation* | | | | | |
| **mirBase** | <http://www.mirbase.org/> |  | miRNA sequence and annotation database |  | Graphic user interface/Web based |
| **Mirsnpscore** | <http://www.bigr.medisin.ntnu.no/mirsnpscore/> |  | miRNA database | Enlists SNPS computationally predicted to effect miRNA target sites |  |
| **MirSNP** | <http://cmbi.bjmu.edu.cn/mirsnp> |  | mRNA Database |  |  |
| **microRNA.org** | <http://www.microrna.org/> |  |  | Contains experimentally observed expression patterns and predicted downregulation scores |  |
| **PolymiRTS database** | <http://compbio.uthsc.edu/miRSNP/> |  | Recommended | Database of variations found in predicted and experimentally verified miRNA sites |  |
| **Patrocles** | <http://www.patrocles.org/> |  | Recommended | Database of polymorphic miRNAs |  |
| ***Changes in Gene Expression*** | | | | | |
| *eQTL resources* | | | | | |
| **eQTL Explorer** | <http://web.bioinformatics.ic.ac.uk/eqtlexplorer/> |  |  |  | Graphic user interface/Web based |
| **eQTL Viewer** | <http://statgen.ncsu.edu/eQTLViewer/> |  |  |  |  |
| **FastMap** | <http://cebc.unc.edu/fastmap.html> |  | Non-functional link |  |  |
| **Lirnet** | <http://www.cs.washington.edu/homes/suinlee/lirnet/> |  |  | Classifies functional SNPs based on a calculated “regulatory potential” depending on gene and SNP details | Matlab;  Uses GenViewer for Windows users. |
| *Changes in splicing* | | | | | |
| **ESEFinder** | <http://rulai.cshl.edu/tools/ESE> |  | Finds exonic splicing enhancer in an input sequence |  | Graphic user interface/Web based |
| **ESRSearch** | <http://esrsearch.tau.ac.il/> |  |  | Finds exonic splicing regulatory elements in an input sequence |  |
| **FAS ESE** | <http://genes.mit.edu/fas-ess/> |  | Finds exonic splicing silencers in an input sequence |  |  |
| **PESX** | <http://cubweb.biology.columbia.edu/pesx/> |  |  | Finds putative exonic silencers and enhancers |  |
| **Rescue ESE** | <http://genes.mit.edu/burgelab/rescue-ese/> |  | Finds exonic splicing enhancer in an input sequence | Provides species filter |  |
| **Human Splice Finder (HSF)** | <http://www.umd.be/HSF/> |  | Highly recommended. Accepts various input formats. | To analyse exonic and intronic mutations causing potential splicing defects | Graphic user interface/Web based |
| **ssSNPTarget** | <http://variome.kobic.re.kr/ssSNPTarget/> |  | Recommended | Compiles Splicing defects/influences caused due to SNP events |  |
| **SNPinfo** | [http://snpinfo.niehs.nih.gov](http://snpinfo.niehs.nih.gov/) |  | Compedium of various useful tools |  | **Web link** |
| *Changes in polyadenylation signals* | | | | | |
| **PolyApred** | <http://www.imtech.res.in/raghava/polyapred/> |  | *In silico* prediction of polyadenylation signals |  | Graphic user interface/Web based |
| **Polyadq** | <http://rulai.cshl.edu/tools/polyadq/> |  | *In silico* detection of Polyadenylation signals |  |  |
| **Mfold** | <http://www.bioinfo.rpi.edu/applications/mfold> |  | Recommended |  |  |
| **pknotsRG** | <http://bibiserv.techfak.uni-bielefeld.de/pknotsrg> |  | Recent | Found in the RNA studio |  |
| **Bielefeld Bioinformatics Server** (**BiBiServ)** | [http://bibiserv.techfak.uni-bielefeld.de](http://bibiserv.techfak.uni-bielefeld.de/) |  | Resource of Bioinformatic tools |  | **Web link** |
| **ERPIN ( Easy RNA Profile IdentificatioN)** | <http://tagc.univ-mrs.fr/erpin/> |  |  | Identification of RNA Motifs and secondary structure | Graphic user interface/Web based |
